# Postoperative Pulmonary Hypertension after Primary Abdominal Wall Closure for Small Omphalocele: A Case Series

**DOI:** 10.1055/a-2937-1779

**Published:** 2026-08-24

**Authors:** Seitaro Kosaka, Takanori Ochi, Shuko Nojiri, Tadaharu Okazaki, Go Miyano

**Affiliations:** 1 Department of Pediatric General and Urogenital Surgery 73362 Juntendo University School of MedicineBunkyoTokyoJapan; 2 73362 Medical Technology Innovation Center, Juntendo University School of MedicineBunkyoTokyoJapan; 3 Department of Pediatric SurgeryJuntendo University Urayasu HospitalUrayasuChibaJapan

**Keywords:** pulmonary hypertension, primary closure, small omphalocele

## Abstract

Primary closure is a low-risk option for small omphaloceles (SOs). However, despite the absence of liver herniation and limited volume of herniated viscera, we encountered two cases of postoperative pulmonary hypertension (PH) and respiratory failure after closure for SO. This report discusses the safety of primary closure and potential factors that may be associated with postoperative PH in infants with SOs.

Cases 1 and 2 presented with SOs without chromosomal abnormalities or liver herniation (gestational ages 36 and 37 weeks, birth weights 3,650 and 2,577 g, and defect sizes 4 and 2 cm, respectively). Prenatal ultrasonography showed no evidence of oligohydramnios in either case, and chest radiography was negative for chest wall deformity at birth. Primary closure was performed within 24 hours of birth after stabilization, defined as venous blood gas values without hypoxia/CO _2_ retention, and echocardiographic confirmation of no PH. Arterial blood gas was assessed after abdominal wall closure and demonstrated critical respiratory failure with CO _2_ retention, and both infants developed postoperative PH. However, low Apgar scores were noted (at 1 minute: 4 and 6 and at 5 minutes: 6 and 6 in Cases 1 and 2, respectively). One infant died from refractory PH, but the other responded to treatment. Even in SO, preoperative respiratory reserve, including information reflected by Apgar scores, may be carefully considered when determining the timing and feasibility of primary closure.

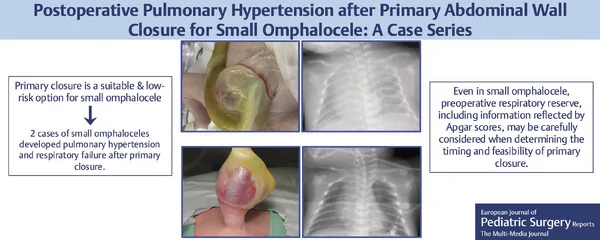

## Introduction

The clinical outcomes of omphaloceles are strongly influenced by associated congenital anomalies or chromosomal abnormalities. Even in isolated omphaloceles, respiratory failure accompanied by pulmonary hypertension (PH) can occur, which may worsen the overall prognosis. 1 PH is frequently observed in infants with giant omphaloceles (GOs), defined as an abdominal wall defect of >4 cm. 2 Furthermore, owing to increased intra-abdominal pressure and upward displacement of the diaphragm, PH may follow abdominal closure, 3 even when staged closure techniques are employed to minimize intra-abdominal pressure. In contrast, small omphaloceles (SOs) typically involve a small volume of herniated viscera and a limited abdominal wall defect, 1 2 making primary closure a suitable and low-risk option. Postoperative PH and respiratory failure are therefore considered rare complications of primary closure for SO. Herein, we describe two cases of postoperative PH in infants who underwent primary closure for SO.

In the context of these cases, we discuss the safety of primary closure and potential factors that may be associated with postoperative PH in infants with SOs. For the purposes of this discussion, SO is defined as an abdominal wall defect ≤ 4 cm with <75% of the liver contained within the sac, based on the criteria established at the Children's Hospital of Philadelphia. 2 This definition was used because assessment based solely on the diameter of the abdominal wall defect may be inadequate, as a large volume of viscera can herniate through a narrow opening. 4

## Case Report

**Fig. 1:**
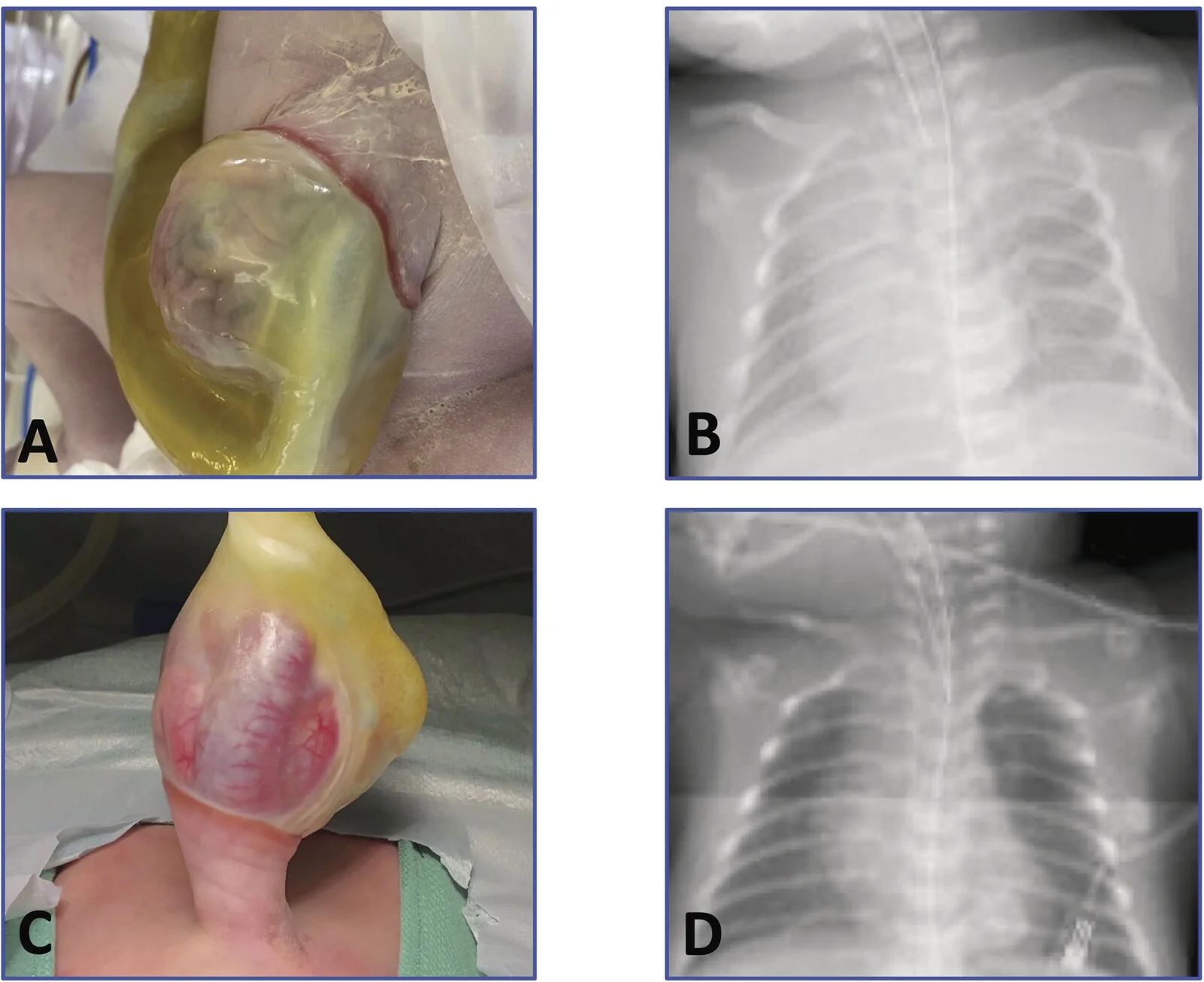
Intraoperative findings and postoperative chest radiograph in Cases 1 and 2. ( **A** ) Case 1 had a 4-cm defect with omphalocele sac containing only a small section of bowel. ( **B** ) Postoperative chest radiographs of Case 1. ( **C** ) Case 2 had a 2-cm defect with a narrow neck, and the omphalocele sac contains only a small section of bowel. ( **D** ) Postoperative chest radiograph of Case 2.

Both cases were referred to our hospital following a prenatal diagnosis of omphaloceles, and prenatal ultrasonography showed no evidence of oligohydramnios in either case. The infants, Case 1 and Case 2, were born at 36 and 37 weeks of gestation, with birth weights of 3,650 and 2,577 g, respectively ( Table 1 ). In both cases, the omphalocele sac contained only a small section of bowel, without liver herniation. The size of the defect was 4 cm in Case 1 and 2 cm, with a narrow neck, in Case 2 ( Fig. 1A, C ). Chest radiography at birth showed no evidence of chest wall deformity or respiratory abnormalities ( Fig. 1B, D ). Perinatal assessment performed immediately after birth revealed Apgar scores of 4 and 6 at 1 minute and 6 and 6 at 5 minutes in Cases 1 and 2, respectively; however, neither patient required intubation.

**Table 1:** Summary of the two cases that developed pulmonary hypertension after primary closure

	Case 1	Case 2
Clinical characteristics		
Gender	M	F
Mode of delivery	Cesarean section	Vaginal delivery
Defect size (cm)	4.0	2.0
Birth weight (g)	3,650	2,577
Gestational age (wk)	36	37
1-min Apgar	4 (A0, P1, G1, A1, R1)	6 (A0, P2, G2, A1, R1)
5-min Apgar	6 (A1, P1, G2, A1, R1)	6 (A0, P2, G2, A1, R1)
Day of primary closure	0	0
Operative duration (min)	92	90
Intraoperative fluid balance (mL)	90	65
Outcome	Survived	Died
Arterial blood gas during primary closure		
pH a	7.305–7.156	7.310–7.210
P/F ratio (PaO _2_ /FiO _2_ ) a	370.4–120.0	270.4–96.8
PaCO _2_ (mm Hg) a	51.9–67.9	45.3–66.6
Lactate (mmol/L) a	1.47–1.59	1.32–1.92

Abbreviations: F, female; M, male.

a Data are presented as preclosure–postclosure values.

After this initial assessment, the infants were admitted to the neonatal intensive care unit (NICU). Both infants met the stabilization criteria, with venous blood gas values within the neonatal reference ranges (pH: 7.21–7.45, pO _2_ > 30 mm Hg, pCO _2_ < 60 mm Hg, and HCO _3_^−^ 22–30 mmol/L 5 ), and echocardiography showed no evidence of cardiac abnormalities or PH (i.e., no flattening of the interventricular septum and/or a tricuspid regurgitant jet with an estimated right ventricular pressure of >40 mm Hg 6 ). The infants then underwent surgery for SO within 24 hours after birth. Both infants were intubated under general anesthesia, an arterial line was placed for blood gas monitoring, and mechanical ventilation was adjusted intraoperatively based on saturation and end-tidal CO _2_ (EtCO _2_ ) monitoring to maintain CO _2_ levels within the physiological range of 35 to 45 mm Hg. Primary closure was performed by reducing the herniated viscera into the abdominal cavity and closing the peritoneum, abdominal fascia, and skin after confirming a tension-free procedure. Arterial blood gas was assessed before and after abdominal wall closure and demonstrated critical respiratory failure with CO _2_ retention and mild lactate elevation after closure ( Table 1 ). Immediately after primary closure, EtCO _2_ increased from 49 to 63 mm Hg in Case 1 and from 43 to 63 mm Hg in Case 2, requiring an increase in ventilatory peak inspiratory pressure from 14 to 18 cmH _2_ O and from 12 to 18 cmH _2_ O, respectively. (Detailed ventilatory settings are shown in Supplementary Table S1 , available in online version only). These findings suggested reduced ventilatory compliance after abdominal closure, likely related to increased intra-abdominal pressure. Regarding fluid management, intraoperative fluids were administered based on standard neonatal anesthetic management to maintain hemodynamic stability, with no excessive fluid loading after closure and no major blood loss. No clear signs of cardiopulmonary compromise were observed intraoperatively before closure.

Postoperatively, the infants were transferred back to the NICU, where echocardiography performed to evaluate the respiratory failure confirmed a diagnosis of PH. Accordingly, inhaled nitric oxide was initiated, and the ventilatory mode was changed to high-frequency oscillation in both cases. Dopamine and dobutamine were administered for circulatory support, sodium bicarbonate was used to correct acidosis, and fentanyl was administered for sedation.

In Case 1, the patient responded to treatment and recovered from PH on postoperative day 1. In contrast, Case 2 did not respond adequately to these interventions and developed bilateral pneumothorax on postoperative day 2, which further compromised cardiopulmonary function. The patient's condition gradually deteriorated thereafter, resulting in death on postoperative day 14.

## Discussion

Similar postoperative cardiopulmonary deterioration has been described in other neonatal surgical conditions, including gastroschisis, in which abdominal wall closure may increase intra-abdominal pressure, elevate the diaphragm, and impair respiratory mechanics. However, PH appears to be more specifically associated with omphalocele than with neonatal abdominal wall closure in general. A direct comparative study demonstrated that patients with omphalocele were 7.78 times more likely to develop PH than those with gastroschisis (95% confidence interval [5.81, 10.41]). 7 This finding suggests that PH in omphalocele is not merely a nonspecific consequence of abdominal surgery or increased intra-abdominal pressure—it may also reflect omphalocele-specific abnormalities in lung and pulmonary vascular development. Abnormal pulmonary vascular development may increase pulmonary vascular resistance and contribute to the development of PH. 2

Infants with GO exhibit a distinct thoracic configuration: upward displacement of the liver and decreased intra-abdominal pressure prevent normal molding of the lower thoracic cage, resulting in an inward collapse of the lower rib cage during respiration. 8 Furthermore, in infants with GO, the rectus abdominis muscles attach laterally to the costal margins of the ribs, contributing to greater caudal rib decline and chest wall narrowing. 8 Consequently, prenatal lung growth in GO is impaired as part of an in utero deformation sequence. 9 PH values of 37 to 57% have been reported in infants with GO, particularly among those with liver in the sac. 1 2 10 In another study comparing GO and non-GO, it was found that infants with GO have a significantly greater severity of PH 11 ; however, postoperative PH after primary closure has not been specifically examined in infants with SO as a distinct subgroup.

In the present report, we describe two infants with SO who developed respiratory failure and PH after primary closure, despite apparent preoperative cardiopulmonary stability and the absence of pulmonary hypoplasia or chest wall deformity.

Thorough clinical evaluation is essential for ensuring neonatal health, particularly in infants with congenital abnormalities, such as omphaloceles. The Apgar score is used to assess neonatal status at 1 and 5 minutes after birth, based on five parameters: appearance, pulse, grimace, activity, and respiration. 12 Accordingly, the low appearance and respiration subscores at 1 and 5 minutes, as observed in both infants in this study, may reflect transient perinatal compromise, including acidosis, poor perfusion, or early cardiorespiratory insufficiency. 13 Although these infants subsequently appeared clinically stable once the initial adaptation was complete, the early compromise may have reflected limited pulmonary and cardiovascular reserves and subclinical pulmonary vascular maladaptation. Primary closure can acutely increase the intra-abdominal pressure and displace the diaphragm cephalad, which decreases lung compliance and functional residual capacity, and worsens ventilation–perfusion mismatch, as suggested by CO _2_ retention after abdominal closure. In addition, worsening acidosis and a slight increase in lactate after abdominal closure in both cases may have reflected transient impairment of systemic perfusion associated with increased intra-abdominal pressure after primary closure. This additional physiological burden may exceed the limited respiratory reserve even in SOs with a small volume of herniated viscera and a limited defect size. In vulnerable infants, hypoxemia after abdominal closure may trigger pulmonary vasoconstriction, ultimately precipitating PH.

In addition, perioperative factors, including general anesthesia, positive-pressure ventilation, and changes in hemodynamics and pulmonary vascular tone, may also have contributed to respiratory failure and PH. 14 15 Postoperative respiratory failure and PH after primary closure in SO may be multifactorial rather than attributed to a single factor.

Various therapies, such as extracorporeal membrane oxygenation, have been reported to treat PH in infants with omphalocele; nevertheless, no standardized management strategy exists, and the mortality rate remains high. 16 Therefore, optimizing perioperative management to prevent PH onset is essential for reducing morbidity and mortality in infants with omphaloceles, including SOs. The two infants in this report initially met the stabilization criteria, supporting progression to primary abdominal closure. However, both had low Apgar scores and later developed PH, illustrating that immediate primary closure after birth may still carry a risk of postoperative PH despite apparent clinical stability and the absence of pulmonary hypoplasia. The fetoneonatal transition is a critical period of cardiopulmonary adaptation after birth, characterized by rapid and complex physiological changes in respiratory and cardiovascular functions. 17 Although this transition is smoothly completed in most term infants, some experience delayed transition, and 5 to 10% have an underlying pathology. 18 Therefore, careful monitoring during this period is essential to promptly identify and manage infants who do not transition successfully. The potential benefit of delaying abdominal closure beyond the immediate postnatal transition period requires further investigation.

We recognize that reliance on conventional indicators of clinical stability may lead to an underestimation of the degree of limited respiratory reserve; therefore, preoperative respiratory reserve, including information reflected by Apgar scores, may be carefully considered when determining the timing of abdominal closure.

The limitations in the interpretation of these cases should be acknowledged. As this observation was based on only two cases, definitive conclusions cannot be drawn regarding the association between low Apgar scores and postoperative PH. In addition, baseline heterogeneity between the two patients, including differences in birth weight, PaO _2_ /FiO _2_ ratio, PaCO _2_ , and Apgar scores, further limits comparability. Further studies with larger and more homogeneous cohorts are needed to validate this observation.

In conclusion, even in SO, preoperative respiratory reserve, including information reflected by Apgar scores, may be carefully considered when determining the timing and feasibility of primary closure.

## Data Availability

The datasets supporting the findings of this study are available by emailing the corresponding author.

## Notes

Importance for the Pediatric Surgeon Pulmonary hypertension and respiratory failure could occur after primary closure even in small omphaloceles, despite the absence of liver herniation and limited volume of herniated viscera, and low Apgar scores in infants with small omphalocele may signal limited cardiopulmonary reserve and increased risk of postoperative pulmonary hypertension.

## Declaration of GenAI Use

During the writing process of this manuscript, the authors did not used any AI tools to assist with manuscript preparation.
