# Supplementary material for: Postoperative Pulmonary Hypertension after Primary Abdominal Wall Closure for Small Omphalocele: A Case Series
Source: European J Pediatr Surg Rep. 2026 Aug 24;14(1):e45–9. doi: 10.1055/a-2937-1779 (PMC13503023; doi:10.1055/a-2937-1779)
Supplement: Supplementary file 1 [file 10-1055-a-2937-1779_29414386.pdf]

Thieme

Supplementary Table S1. Summary of ventilator settings and $EtCO_2$ before and after closure

Case	*Timing*	PIP (cmH_2O)	PEEP (cmH_2O)	FiO_2	RR (/min)	$EtCO_2$ (mmHg)
**1**	Before closure	14	5	0.40	30	49
	After closure	18	5	0.60	30	63
2	Before closure	12	5	0.40	40	43
	After closure	18	5	0.60	40	63

PIP, peak inspiration pressure; PEEP, positive end-expiratory pressure; FiO_2, fraction of inspired oxygen; RR, respiratory rate; $EtCO_2$, end-tidal CO_2

Both patients were ventilated using pressure-controlled ventilation during surgery.
